# Hyperthermia-Induced Disruption of Functional Connectivity in the Human Brain Network

**DOI:** 10.1371/journal.pone.0061157

**Published:** 2013-04-08

**Authors:** Gang Sun, Shaowen Qian, Qingjun Jiang, Kai Liu, Bo Li, Min Li, Lun Zhao, Zhenyu Zhou, Karen M. von Deneen, Yijun Liu

**Affiliations:** 1 Department of Medical Imaging, Jinan Military General Hospital, Jinan, Shandong, People's Republic of China; 2 McKnight Brain Institute, University of Florida, Gainesville, Florida, United States of America; Zhejiang University School of Medicine, China

## Abstract

**Background:**

Passive hyperthermia is a potential risk factor to human cognitive performance and work behavior in many extreme work environments. Previous studies have demonstrated significant effects of passive hyperthermia on human cognitive performance and work behavior. However, there is a lack of a clear understanding of the exact affected brain regions and inter-regional connectivities.

**Methodology and Principal Findings:**

We simulated 1 hour environmental heat exposure to thirty-six participants under two environmental temperature conditions (25°C and 50°C), and collected resting-state functional brain activity. The functional connectivities with a preselected region of interest (ROI) in the posterior cingulate cortex and precuneus (PCC/PCu), furthermore, inter-regional connectivities throughout the entire brain using a prior Anatomical Automatic Labeling (AAL) atlas were calculated. We identified decreased correlations of a set of regions with the PCC/PCu, including the medial orbitofrontal cortex (mOFC) and bilateral medial temporal cortex, as well as increased correlations with the partial orbitofrontal cortex particularly in the bilateral orbital superior frontal gyrus. Compared with the normal control (NC) group, the hyperthermia (HT) group showed 65 disturbed functional connectivities with 50 of them being decreased and 15 of them being increased. While the decreased correlations mainly involved with the mOFC, temporal lobe and occipital lobe, increased correlations were mainly located within the limbic system. In consideration of physiological system changes, we explored the correlations of the number of significantly altered inter-regional connectivities with differential rectal temperatures and weight loss, but failed to obtain significant correlations. More importantly, during the attention network test (ANT) we found that the number of significantly altered functional connectivities was positively correlated with an increase in executive control reaction time.

**Conclusions/Significance:**

We first identified the hyperthermia-induced altered functional connectivity patterns. The changes in the functional connectivity network might be a possible explanation for the cognitive performance and work behavior alteration.

## Introduction

Hyperthermia is a very common environmental factor in many work places, such as a product manufacturing plant, coal mine military operation, firefighting and outdoor sports. The abilities of work behavior and safety were reported to be significantly affected by environmental heat stress [1]–[3]. Prolonged exercise or work during hyperthermia could elevate skin temperature, core temperature and brain temperature rapidly and cause heat-induced fatigue expressed as time to exhaustion and increased rating of perceived exertion (RPE), and further impair the cardiovascular system, neurohormonal activity and muscle metabolism [4], [5]. Besides the physiological aspects, hyperthermia could also cause central nervous system (CNS) abnormalities [6]. For example, it has been reported that exercise capacity was decreased during passive hyperthermia accounted much by CNS motor drive rather than peripheral muscle failure [7]–[9]. Moreover, passive hyperthermia impairs cognitive function and behavior, such as vigilance performance, tracking performance and simple performance [10], [11]. It was also found that hyperthermia decreased memory capacity in both working memory and short-term memory, implying abnormal activity in the frontal lobe [12]. Recording the electroencephalograph (EEG) in patients with cancer during passive hyperthermia, Gaoua et al. [11] assessed the effects of passive heating upon attention and memory task performance and found that there was impairment in working memory with heat exposure without alteration in attention processes.

During recent years, growing proofs of altered brain electrical activity have been obtained to provide more compelling evidence for altered cognitive performance and behavior during hyperthermia. Dubois et al. [13] reported a reduced EEG spectrum for high frequencies of the EEG spectrum which was reduced in patients with cancer during passive hyperthermia. Similarly, Nielsen et al. [14] found that compared with a cool (19°C) environment, a hot (42°C) environment elevated the α/β index of the frontal lobe. More recently, Sun et al. [15] investigated the effect of hyperthermia on pre-attentive processing by recording the mismatch negativity (MMN) components of event-related potentials (ERPs) and found that MMN declined significantly in the HT group compared with the NC group after 50°C heat exposure for 1 h, implying damage from passive hyperthermia on pre-attentive processing. However, the neuro-electricity phenomenon was not sufficient to elucidate the heat-affected regions or multi-region connectivity.

In consideration of a specific regional response to hyperthermia, elevated body temperature would activate the thermoregulation mechanism (autonomic and behavioral thermoregulatory) [16]–[18]. From the perspective of function segregation, previous fMRI studies assessed discrete activated human brain regions correlated with thermal regulation and sensation to environmental heat exposure, involving an intact preoptic area and anterior hypothalamus, as well as the primary and secondary somatosensory, the amygdala, and the dorsomedial hypothalamus [16], [17]. For example, Kanosue et al. [19] found that bilateral amygdalar activity was associated with thermal exposure intensity. However, the activations of these life-support regions rather than cognitive-support regions were not sufficient to elucidate wide-range cognitive and behavioral alteration during passive hyperthermia, awaiting addressing the functional state of a high-level CNS, such as activations in the frontal lobe, parietal lobe, temporal lobe and so on. During last few decades, functional connectivity which refers to the temporal correlation between neurophysiological measurements made in different brain areas during the resting state [20]–[22], has been widely used to investigate the dysfunctional integration of a set of brain regions in brain diseases such as Alzheimer's disease, schizophrenia, depression, drug addiction, attention deficit hyperactivity disorder (ADHD) and so on [23]–[25]. Functional connectivity, as a method of functional integration, concentrated on alteration in multi-regional connectivities of human brain functional networks, and might provide evidence for wide-range altered cognitive and behavioral functions.

Therefore, the aim of the present study was to examine the influences of passive hyperthermia on human brain functional connectivity patterns. A further aim was to examine whether the connectivity alterations were associated with behavioral performance and physiological changes. We hypothesized that altered functional connectivity patterns of high-level nervous regions, such as the prefrontal lobe, would occur in response to heat exposure and the alteration would be related to behavioral performance.

In the present study, we will investigate the effects of hyperthermia on the functional connectivity patterns in the brain by recording BOLD-fMRI, in which functional connectivity based on a preselected seed ROI, specifically the PCC/PCu, was computed for both groups. In addition, to avoid the limitation of the biased ROI-based method, we further divided the entire brain into 90 regions and analyzed the correlations between each pair of these regions. Then, we identified the significantly altered functional connectivity patterns relative to the NC group.

## Materials and Methods

### Participants and experiments

Thirty-six healthy right-handed college students (male; 21.5±1.82 years, ranging from 18 to 23 years) participated in this study. All of the participants were free of any neurological or psychiatric disorders and never participated in any fMRI experiments before and there was no significant difference in age and education level between both groups. The research protocol was approved by the Jinan Ethical Committee, and all of the participants had given written informed consent, as outlined in the PLOS consent form, to publication of their photographs.

**Figure 1:**
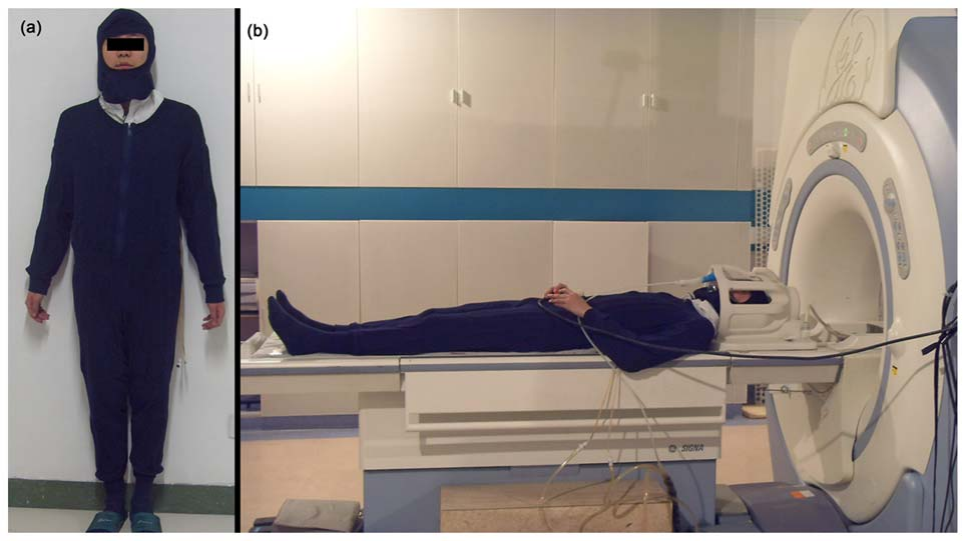
One participant wearing the thermal suit before the entrance into the environmental chamber (a) and before fMRI scanning (b). The double-layer suit, including pants, shirts and hat, was made of flexible fabric with a series of embedded pipelines. During the fMRI scan, the pipelines were connected to the subsidiary temperature control device, which can regulate the temperature of the water circulating in the pipelines. Also, a respiration hood was connected to an air container and air temperature controller. The air was heated to the preset temperature before it was exported to the respiration hood.

The thirty-six participants were randomly divided into two groups, an NC group (n = 18; 21.9±1.67 years, ranging from 18 to 25 years) with a 40-minute exposure to a temperature of 25°C and relative humidity (rH) of 60%, and an HT group (n = 18; 21.1±1.55 years, ranging from 19 to 23 years) with a 40-minute exposure at 50°C and 60% rH in an environmental chamber. The temperature and humidity inside the chamber could be set arbitrarily within a range of −10°C to 55°C and 10% rH to 90% rH using two temperature controllers (NTFA-18, Gree) and two humidifiers (LH8809, Weiran). Once the temperature inside the chamber room dropped below the designated 50°C or 25°C outside of the allowed range, the electronic heaters would quickly turn on automatically. Once the temperature reached beyond 50°C or 25°C, the heater would turn off immediately. Thus, the temperature was kept constant at the designated 50°C or 25°C at the expected range of ±1°C. All participants wore a thermal lab-suit covering the whole body as well as the head. The suit was designed with a soft embedded pipeline in which the hot water would circulate to simulate environmental heat during fMRI scanning. After the 40-minute heat exposure inside the chamber, the participants were taken to the MRI room for scanning. During the scanning, the pipeline in the suit was connected to a subsidiary water temperature control device (OWK-C, Germany Ouchida Int'l Group Limited), with the hot water circulating throughout the pipeline. To optimally simulate the heated environment, participants wore a respiration hood in which the hot air circulated with the air temperature controlled by the same device. The hot water and air were both designated at 50°C in the HT group and at 25°C in the NC group. During the resting-state scan, the participants were instructed to lie quietly and remain motionless with their eyes open, and to not engage in any mental activity (Fig. 1). The resting-state fMRI scan took about 20 minutes, thus, combined with the time in the environmental chamber, each participant endured heat exposure for approximately 1 hour. We recorded the heart rate and respiration rate intermittently using the MR scanner continuous measurement. Rectal temperatures of all of the participants were recorded before entering the environmental chamber and after the fMRI scan using a rectal temperature logger (ZWJ-2, Tianjin Honour Instruments Co., Ltd), since rectal temperature has been regarded as a good analog of brain temperature and core body temperature, it was used to record core body temperature changes during hyperthermia in several previous studies [26]–[28]. To detect dehydration during environmental heat exposure, net weight of each participant was measured using an electronic weightometer (Xiangshan, EF901) before and after the experiments in both groups, and the weight loss was then calculated. No water drinking was allowed in the experiments. All the participants had rights to discontinue the experiment if they felt very uncomfortable. After the HT scans, the participants reported their affective sensations about the thermal condition with a three point scale: very uncomfortable, uncomfortable, neutral. The images of participants who felt very uncomfortable would be discarded.

To further identify impaired cognitive performance and work behavior during passive hyperthermia, we performed a modified version of the ANT proposed by Fan et al. [29]. As depicted in Figure S1 in the supplementary part, three cue conditions (no cue, center cue and spatial cue) and two target types (congruent and incongruent) were used to uncover three attentional networks in a single task. Each trial began with the presentation of a fixation cross followed by one of the three cue conditions with a duration of 200 ms. After a time interval of 300–11800 ms, the target was presented 1.06°above or below the fixation point. The participant identified the direction (left or right) that the target pointed to by pressing a button as quickly as possible. The target display disappeared until a response was made or after 2000 ms with no response, while the fixation point remained on the screen for a variable duration of 3000–15000 ms. One run contained 36 trials and each participant was tested for 6 runs and the protocol took about 35 min. To measure the efficiency of three attentional networks, reaction time (RT) was calculated for different cues and target conditions:
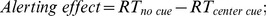


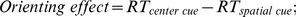


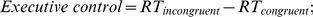



Details about the ANT procedure can be seen in the paper by Fan et al. [29] and our previous study [30].

### Data acquisition

The resting-state scans were obtained for all of the participants using a GE Signa 1.5 T scanner (General Electric, Milwaukee, Wisconsin). The participants lay in a supine position with their heads fixed in place by foam pads to minimize head translation and rotation movements. Each scan consisted of 200 EPI functional volumes with the following parameters: TR = 2000 ms, TE = 40 ms, flip angle (FA) = 90°, number of slices = 29, matrix = 64×64, field of view (FOV) = 24×24 cm^2^, thickness/gap = 4/0 mm, acquisition voxel size = 3.75×3.75×4 mm^3^. Additionally, a high resolution T1-weighted sequence was obtained: 115 slices, TR = 11.1 ms, TE = 4.9 ms, slice thickness = 1.4 mm, FOV = 24×24 cm^2^, FA = 20°.

### Data preprocessing

Preprocessing of the functional data was performed with SPM8 package (http://fil.ion.ucl.ac.uk/spm) implemented by using MATLAB, version7.10 (MathWorks, Natick, Mass). The first ten volumes were discarded. All of the images were corrected for intra-volume acquisition time offsets between slices and were then realigned to the first volume image for inter-scan head movement correction. Then, the functional images were normalized to the standard Montreal Neurological Institute (MNI) space and resampled to 3×3×3 mm^3^. Subsequently, the images were spatially smoothed with a 4×4×4 mm^3^ full width at half maximum Gaussian kernel to minimize spatial noise. Finally, Resting-State fMRI Data Analysis Toolkit v1.7 (REST, http://www.restfmri.net/) [31], was used to remove the linear trend and for temporally band-pass filtering (0.01∼0.08 Hz) the data, thus reducing the low-frequency drift and high-frequency physiological noise. Several sources of spurious variance were removed from the data via linear regression: signals from white matter, global mean signal, cerebrospinal fluid and head motion. Due to the excessive head motion artifact and thermal discomfort report, two participants in the HT group reported unbearable thermal discomfort and their scans were terminated, and one NC group participant and one HT group participant had greater than 1 mm maximum displacement in any of the x, y, z directions or greater than 1°angular rotation and their images were discarded.

### ROI selection and functional connectivity analysis of the entire brain

Previous studies demonstrated that the PCC/PCu plays a key role in the brain, exhibiting a high metabolic rate [32]. In addition, the PCC provides a key hub for overlapping connections between itself, the medial temporal lobe and inferior parietal lobe [33]. With respect to clinical research, abnormal PCC/PCu connectivity was found to be associated with several neuropsychiatric disorders, such as Alzheimer' s disease, schizophrenia, mesial temporal lobe epilepsy and so on [23], [33], [34]. In the field of human brain network research, key hub region has the highest centrality and density of connectivities [35], [36]. Therefore, the correlation analysis based on a seed reference in the PCC/PCu may provide important information about alteration during hyperthermia. The PCC/PCu (MNI coordinates, [−2, −54, 27], r = 10 mm) was then chosen as a spherical ROI using the MarsBaR software package (http://marsbar.sourceforge.net/). The mean time series was obtained by averaging the time series of all of the voxels within the ROI. A correlation analysis was carried out between the ROI seed and the whole brain in a voxel-wise manner. A functional connectivity map for each participant was obtained and further transformed to a z functional connectivity (zFC) map by Fisher's r-to-z transformation to improve normality. The zFC maps of each participant were entered into a random effects one sample *t*-test to determine the regions showing significant connectivity to the PCC/PCu under a single voxel threshold of *p*<0.05 and cluster size of at least 810 mm^3^ using AlphaSim correction by Monte Carlo simulation (parameters were: FWHM = 4 mm, with a mask of the whole brain). The zFC maps were further entered into a random-effects two-sample *t*-test to identify regions showing significant differences in the PCC/PCu connectivity between the HT and NC groups. Significant between-group differences were determined again with a single voxel threshold of *p*<0.05 and cluster size of at least 810 mm^3^ using the AlphaSim correction.

Although PCC/PCu connectivity analysis provided the alteration of key hub connectivities during hyperthermia, the other connectivities between other regions that were not significantly correlated with the PCC/PCu might be ignored on account of methodological limitation. To provide more detailed information about the brain network and its changes under heat stress, we assessed landmark-based functional connectivity throughout the entire brain [37], [38]. Briefly, the functional images were registered with the MNI template and further divided into 90 regions according to a prior AAL previously reported by Tzourio-Mazoyer et al. [39]. The regions and their abbreviations used in the present study can be seen in the supplemental materials (Table S1). The mean time series from each of the 90 regions was calculated by averaging the time series of all of the voxels within that region. Functional connectivity, measured by the Pearson correlation coefficient, was then computed between each pair. The obtained functional connectivity matrix was converted to the zFC matrix using Fisher r-to-z transformation. The zFC matrix of each participant was entered into a random effects one sample *t*-test to determine the brain regions showing significant within-group correlations. More importantly, a random effects two sample *t-*test was applied to these zFC matrices to show significant differences in functional connectivity between the groups. The statistical significance level was determined by the following two criteria: (1) the z values were significantly different from zero within the group at a threshold of *p*<0.05 (one-sample two tailed *t*-test; FDR corrected); (2) the z values were significantly different between the two groups at a threshold of *p*<0.05 (two-sample two-tailed *t*-test; FDR corrected).

Furthermore, to investigate correlations of the changes in the functional connectivity patterns with core temperature changes and dehydration, we also performed a Pearson correlation coefficient between rectal temperature changes, weight loss and the number of significantly altered functional connectivities throughout the whole brain based on AAL parcellation during the HT condition.

## Results

There were no significant differences in age and education level between the HT and NC groups. The average rectal temperature did not differ between the two groups before the experiment (HT *vs*. NC: (37.37±0.36°C) *vs*. (37.35±0.35°C); *p* = 0.903) but it was significantly higher in the HT group than in the NC group after the experiment (HT *vs*. NC: (37.87±0.27°C) *vs*. (37.37±0.34°C); *p*<0.001). No significant weight loss was found in the NC group; apparently, significant weight loss was found in the HT group (0.51±0.18 kg).

### Seed-based functional connectivity using PCC/PCu

#### 1) Within group analyses of the PCC/PCu connectivity

**Figure 2:**
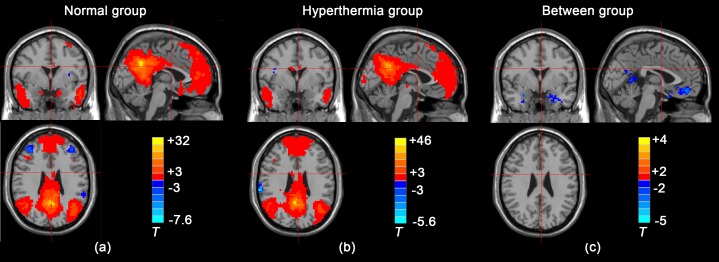
Maps of brain regions showing significant positive or negative correlations with the PCC/PCu in a voxel-wise manner of within-group analysis. (a) NC group, (b) HT group and between-group analysis (c).

Within-group analysis was performed using the REST-v1.7 random-effects one-sample *t*-test showing a number of brain regions that were significantly correlated (positively) or anti-correlated (negatively) with the PCC/PCu in each group. During initial visual inspection, both groups showed a similar functional connectivity pattern. Specifically, the ventral anterior cingulate cortex (vACC), bilateral temporal cortex, medial prefrontal cortex (mPFC), and PCC/PCu were positively correlated with the PCC/PCu. Furthermore, other regions such as the bilateral insula, bilateral supramarginal gyrus, inferior parietal lobule, prefrontal cortex including the superior lateral prefrontal cortex and right inferior frontal gyrus were negatively correlated with the PCC/PCu (Fig. 2(a–b)). The results also showed that the number of voxels that were significantly correlated/anti-correlated with the PCC/PCu in the HT group was fewer than in the NC group. In particular, regions of the mOFC and insula disappeared from the functional connectivity map in the HT group.

#### 2) Altered PCC/PCu connectivity between the HT and NC groups

Between-group difference analysis was performed using the REST-v1.7 random-effects two-sample *t*-test showing significant alteration in the PCC/PCu connectivity that might be induced by hyperthermia. The between-group zFC difference maps demonstrated significant decreases in PCC/PCu connectivity in a number of brain regions. These brain regions included the mOFC, bilateral medial temporal cortex (parahippocampal gyrus and hippocampus), ROI-adjacent PCC/PCu and ACC. On the other hand, regions showing increased correlations with the PCC/PCu were located in the bilateral orbital superior frontal gyrus. Specific cluster locations are depicted in Fig. 2(c) and Table 1.

**Table 1:** Areas with significantly altered functional connectivities with the PCC/PCu between the HT and NC groups.

Region	BA	X	Y	Z	Peak *t* Score	Cluster
mOFC	11	2	39	−16	−3.75	348
ACC	11	−8	29	−6	−2.37	51
left parahippocampal gyrus/hippocampus	36/20	30	−2	−21	−3.2	37
PCC/PCu	23/31	3	−60	21	−3.14	56
right parahippocampal gyrus/hippocampus	30	−18	−24	−18	−4.14	51
**left superior frontal gyrus, orbital part**	**11**	**−24**	**66**	**−9**	**3.31**	**33**
**right superior frontal gyrus, orbital part**	**11**	**12**	**69**	**−6**	**3.93**	**45**

BA, Brodmann's area; (x, y, z) coordinates of the primary peak locations in the space of the Montreal Neurological Institute (MNI) space. Those significant increased correlations were highlighted by bold font while the decreased ones were displayed by normal font.

### Functional connectivity between AAL-division regions

#### 1) Within group functional connectivity

**Figure 3:**
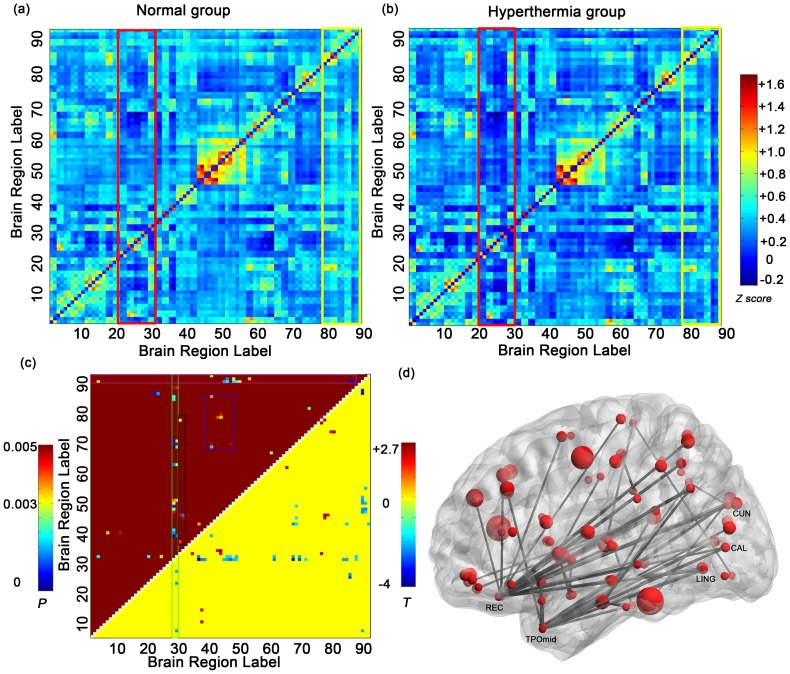
Results of functional connectivity analysis throughout the entire brain. (a–b) Mean zFC matrices of both groups throughout the entire brain divided into 90 regions by the AAL atlas. Visually different correlations between both groups mainly focused on the prefrontal regions (red rectangle) and temporal regions (yellow rectangle). (c–d) Significant correlations throughout the whole brain between both groups are depicted in the matrix figure (c) and brain network figure (d). The top-left triangle in Fig. 3 (c) exhibits the p value of the significant correlations and the bottom-right one exhibits the T value of the corresponding correlations. The rectangles in green, pink, blue and black represent significant altered correlations. Details can be seen in the Result part.

Both groups showed a rather similar functional connectivity pattern. The majority of significant correlations between the inter-hemispheric symmetrical regions are depicted in Fig. 3(a–b) around the diagonals. In addition, a set of regions (rectangles in Fig. 3(a–b)) showed visible differences in the correlations between groups which mainly focused on the prefrontal regions (mainly within the AAL-region 20 to AAL-region 30, red rectangle in Fig. 3(a–b)), and temporal regions (mainly within AAL-region 80 to AAL-region 90, yellow rectangle in Fig. 3(a–b)).

#### 2) Altered functional connectivity between both groups

In all of the 4005 functional connectivities throughout the entire brain, 65 inter-regional correlations showed significant differences between the HT group and the NC group (*p*<0.05, FDR corrected), including 50 decreased correlations and 15 increased correlations. Details can be seen in Fig. 3(c–d) and Table S2 in the supplementary materials.

Compared with the NC group, the altered correlations were mostly concentrated in the prefrontal cortex, temporal lobe and occipital lobe. Among them, there were 26 decreased correlations between the bilateral gyrus rectus (AAL-region27/AAL-region28) and other regions which were focused in the partial frontal cortex, temporal lobe and occipital lobe. Specifically, we found 4 decreased correlations between the bilateral gyrus rectus and part of the frontal lobe including the bilateral superior frontal gyrus, supplementary motor area, medial superior frontal gyrus, and paracentral lobule. Our results also showed 9 decreased correlations between the bilateral gyrus rectus and parietal lobe including the PCC, postcentral gyrus, angular gyrus, PCu, superior parietal gyrus, and 5 decreased correlations between the gyrus rectus and temporal lobe including the hippocampus, superior temporal gyrus, and middle temporal gyrus. The statistical analysis also showed that there were 8 decreased correlations between the gyrus rectus and occipital lobe including the calcarine fissure, cuneus, and lingual gyrus. The altered correlations within the bilateral rectus gyrus are mainly enclosed in the green rectangle depicted in Fig. 3(c).

There were also 23 significantly decreased correlations (partially overlapping the 26 correlations mentioned above) between the temporal lobe (specifically AAL-region 81 to AAL-region 90) and other regions, which mainly focused on the contralateral temporal lobe, frontal lobe, parietal lobe and occipital lobe specifically, including the bilateral rectus gyrus, bilateral lingual gyrus, calcarine fissure, cuneus, middle occipital gyrus and so on. These altered correlations are partially included in the pink rectangle in Fig. 3(c).

Compared with the NC group, 15 increased correlations were found mainly within the limbic system, specifically in the partial prefrontal lobe, temporal lobe, parietal lobe, insula, basal ganglia, and thalamus. Among them, the median cingulate and paracingulate gyri, amygdala, and putamen showed 5 increased correlations with the bilateral insula (AAL-Region 29/AAL-Region 30) which are partially depicted in a black rectangle in Fig. 3(c). Furthermore, there was a set of regions showing 6 increased correlations with the bilateral amygdala including the insula and basal ganglia. In addition, the bilateral thalamus showed 2 increased correlations with the right paracentral lobule. These altered correlations are also shown in the blue rectangle in Fig. 3(c). Details can be found in Table S2.

### ANT test results and correlations with the number of significantly altered functional connectivities

**Figure 4:**
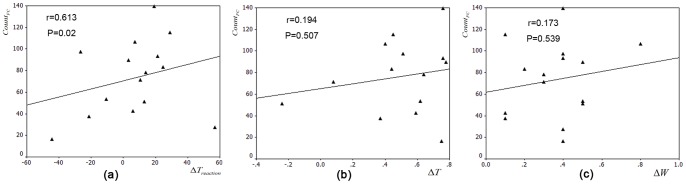
Results of correlation analysis of functional connectivity changes with behavioral performance and physiological changes. (a) Scatter plots of the number of significantly altered functional connectivities (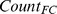) and the increase of executive control reaction time (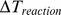).Significant positive correlations were found between 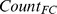 and 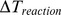. (b–c) Scatter plots of the number of significantly altered functional connectivities (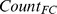) and the differential rectal temperature (b), weight loss (c), but no significant correlations were found.

Consistent with our previous study [30], we did not find significant alterations in the alerting (t(30) = −1.102, *p* = 0.279) or the orienting (t(30) = 0.31, *p* = 0.759) effects between both groups. But the executive control effect showed significant differences (t(30) = 2.747, *p* = 0.01). We further examined the correlations between the increase of executive control reaction time (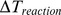) and the number of significantly altered functional connectivities (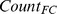) throughout the whole brain (Fig. 4(a)). We found that 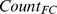 was significantly positively correlated with the 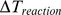 (r = 0.613, *p* = 0.02). In other words, the more altered functional connectivities hyperthermia induced, the longer 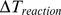 would be in the HT condition.

### Correlations of the alterations of network metrics with rectal temperature and weight loss

In order to primarily explore the potential influences of the elevated core temperature and dehydration on the alteration of the human brain functional connectivity patterns, we examined the correlations between the number of significantly altered functional connectivities throughout the whole brain and rectal temperature change, as well as weight loss in the HT group. Fig. 4(b–c) depicted the correlations between the number of significantly altered functional connectivities (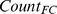) and differential rectal temperature (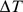) and weight loss (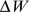). We found that the number of significantly altered functional connectivities (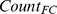) was not significantly correlated with the rectal temperature alteration (r = 0.194, *p* = 0.507), as well as weight loss (r = 0.173, *p* = 0.539).

## Discussion

In the present study, based on stable cerebral blood flow and blood distribution of resting-state during passive hyperthermia [40], [41], we examined the altered functional connectivity network in the human brain. Compared with the NC group, the HT group showed that decreased correlations were mainly focused in the prefrontal cortex, temporal lobe, and occipital lobe and increased correlations were mainly located within the limbic system. Additionally, both methods indicated that the most hyperthermia-affected region was located in the mOFC. We also tried to explore the correlations of the number of significantly altered functional connectivities throughout the whole brain with rectal temperature and weight loss, but failed to obtain significant correlations. More importantly, during the ANT test we found the number of significantly altered functional connectivities throughout the whole brain was positively correlated with the increase of executive control reaction time.

### Altered functional connectivities during passive hyperthermia using ROI

Consistent with previous studies [32], [42], the present study observed regions showing significant connectivities with the PCC/PCu including the mPFC, the PCC, the ACC, the bilateral hippocampus, the inferior temporal cortex, the cuneus, the PCu, and the inferior parietal cortex. These functional connectivities reflect a default mode of brain function and constitute a default mode network (DMN). It was suggested that the DMN might be associated with self-referential aspects of the task [43], episodic memory [44], emotion and anxiety [45], and that abnormal functional connectivity in the DMN may indicate disordered functional coherence and cognitive information transformation and processing [46]–[48].

It has been well known that the PCC/PCu play a key role in the DMN. In the present study, the PCC/PCu showed a decreased correlation with the mOFC, as well as an increased correlation with the bilateral orbital superior frontal gyrus, indicating temporal coherence disturbance between the anterior-posterior cortex and information transformation. In the HT group, the decreased PCC/PCu functional connectivity with the mOFC may suggest disturbed cognitive function and behavior which the correlation supports. Supporting this, previous behavioral and electrophysiological studies identified prefrontal-supporting cognitive function and behavior performance deficits. For example, Racinais et al. [12] proposed abnormal activity in the frontal lobe during the heat stress condition on the basis of the fact that hyperthermia decreases memory capacity in both working memory and short-term memory which are relevant to frontal lobe activity. Moreover, Nielsen et al. [14] considered that alterations of electrical activity in the brain's frontal area changes in parts of the brain were involved in the hyperthermia-associated reduced ability to exercise. Unlike previous studies, the present fMRI study located the altered regions to the so-called mOFC, rather than the frontal lobe. The human brain is a complex network and once its function is disturbed, the corresponding compensatory mechanism will be constructed quickly. Increased correlations between the ROIs and the bilateral orbital superior frontal gyrus may be interpreted as a compensatory reallocation or recruitment of mOFC cognitive resources. This issue awaits further investigation. In addition, the partial medial temporal lobe, specifically the parahippocampal gyrus and hippocampus, were found to have decreased correlation with the PCC/PCu. The parahippocampal gyrus and hippocampus have been identified to be the main brain structures involved in learning, memory and spatial location and were the key nodes within the DMN [23]. Moreover, several axonal tracing studies have reported that neural connectivities between the PCC/PCu and medial temporal lobe, especially in the left parahippocampal gyrus, act as key memory centers [49]–[51]. The decreased hippocampal functional connectivity with the PCC/PCu within the DMN in the present study may suggest abnormal work memory processing during hyperthermia, providing neuroimaging evidence for behavioral findings of impaired working memory during hyperthermia [11], [12].

Besides the decreased inter-regional correlations, the ROI had decreased correlations with the nearby PCC/PCu cortex which the ROI did not completely cover. This result may suggest that there were altered within-region correlations in the PCC/PCu except for the altered between-region correlations with the PCC/PCu, implying abnormal PCC/PCu function, such as self-referential cognitive activity, gathering and evaluating the information about the world or within ourselves as demonstrated in previous studies [32], [42]. Supporting this hypothesis, Hocking et al. [2] administered a range of psychometric tests on cognitive processes and the results markedly indicated deficits in information collection and retention. Furthermore, pre-attentive processing was impaired during passive hyperthermia [15]. This may suggest heat-induced abnormal allocation of attention resources.

### Altered functional connectivities during passive hyperthermia using the correlations throughout the entire brain

To avoid the limited information of the biased ROI-based method, we calculated the functional connectivity throughout the entire brain, offering global distribution of the altered connectivity during hyperthermia. Compared with the NC group, among the 90 regions there were 65 inter-regional functional connectivities that were significantly altered in the HT group, indicating decreased correlations accounting for 77% of the total. This finding was consistent with the alteration of functional connectivity based on the ROI method mentioned above in the present study, suggesting deficits of temporal coherence between brain regions related to neuronal spontaneous activity. These deficits may further disturb inter-regional and intra-regional information processing and transmission, and subsequently, the disturbance would further impair brain function and cognitive performance [2], [11], [52].

Combined with the first seed-based method, the most hyperthermia-affected region was located in the mOFC. Among 65 altered correlations, we further found 50 decreased correlations in which there were 26 significantly decreased correlations between the mOFC (e.g., bilateral gyrus rectus) and other regions, further supporting the finding using the ROI that the decreased ROI-associated correlations were mainly focused on the mOFC (accounting for approximately half of the decreased correlations). Consistent with previous studies [16], [18], these data suggested that hyperthermia-induced connectivity alterations may result from abnormal function of the mOFC. Interestingly, decreased correlations were also found among the temporal lobe, occipital lobe and parietal lobe. This presents our hyperthermia-induced effects on the human brain as disturbed functional connectivities among the anterior-posterior-temporal cortex and implies that hyperthermia-induced brain network alterations may result from the altered correlations between several regions, especially the frontal lobe and temporal lobe, rather than activation or deactivation of several discrete regions [16], [18]. For example, the mOFC showed significantly altered connectivities with a number of regions. It is known that information about skin temperature which projects to the orbitofrontal cortex may be involved with information transmission from the somatosensory cortex and insular somatosensory areas to the orbitofrontal cortex, suggesting a cooperation of several regions for skin temperature information transmission [53]. Moreover, the orbitofrontal cortex shares extensive connections with other association cortices, primary sensory and association cortices, limbic systems, and other subcortical areas. Corticocortical connections include extensive local projections to and from other prefrontal regions, motor, limbic, and sensory cortices. Areas projecting to motor areas are densely interconnected with other prefrontal cortical regions, reflecting integration for executive motor control [54]. This may be a possible explanation for central nervous system fatigue during a hot environment which resulted in a decline in exercise capacity or an elevated rating of perceived exertion (RPE) [7], [8].

Finally, in the HT group, 15 increased correlations were demonstrated mainly within the limbic system, specifically the insula, basal ganglia, and thalamus, as seen with the findings using the ROI in the partial orbitofrontal cortex (bilateral orbital superior frontal gyrus) which had increased correlation with the PCC/PCu. A possible explanation for the altered functional connectivities in the present study might be in some extent accounted by activation or deactivation of discrete regions. Elevated body temperature would activate the thermoregulation mechanism, involving an intact preoptic area and anterior hypothalamus, as well as the insular, primary and secondary somatosensory, the amygdala, and the dorsomedial hypothalamus [16]. Rolls et al. [18] reported that the ventral posterior insular cortex, a part of the somatosensory cortex and ventral striatum were associated with “thermal sensation” and were correlated with the intensity of the thermal stimuli by applying innocuous thermal stimulation to a limited area for a short period. There were also several other studies which reported brain areas including the pregenual cingulate cortex, insula, amygdala, and the primary and secondary somatosensory cortices which were involved in “thermal comfort/discomfort” and behavioral thermoregulatory signals [16], [18], [19], [55], [56]. With the wide heat stimulation area (the whole body) in the present study, heat exposure would cause the participants to feel uncomfortable and become a substantial load for thermoregulation. Thus, it is natural that abnormal functional connectivities were reported in both “thermal sensation” and “thermal comfort/discomfort” associated regions in the present study. Another possible explanation for the altered limbic system connectivity could be that the limbic system is relevant to the control of emotions and mood state in humans [57]–[59] and in particular, there was evidence that heat stress would induce negative effects on the mood state for vigor which was significantly decreased, for fatigue which was significantly increased and for tension which was slightly decreased [26]. The altered functional connectivity of the limbic system obtained in the present study may offer a new perspective in interpreting the mood state alteration associated with environmental heat.

### Relationship between the number of significantly altered functional connectivities and ANT test performance

Importantly, to identify the deteriorated behavior performance and its correlation with the altered functional connectivity network, we copied the ANT procedure performed in our previous study [30], and found the number of significantly altered functional connectivities were positively correlated with the increase of executive control reaction time. The more number of significantly altered functional connectivities hyperthermia induced, the longer the executive control time the participants used. The findings appeared to demonstrate that the executive control performance decline to some extent may be accounted by the severity of the alteration of the functional connectivity patterns during passive hyperthermia. Changes in the functional connectivity network might be a possible explanation for the cognitive and behavioral alteration during passive hyperthermia. It was consistent with previous behavioral studies mentioned above [2], [10], [60], [61], providing more evidence for the decline in hyperthermia-induced human performance.

### Relationship between the number of significantly altered functional connectivities and physiological changes

In the present study, rectal temperature was significantly higher after heat exposure in the HT group, but no significant correlations were found between the number of significantly altered functional connectivities throughout the whole brain and the differential rectal temperature. This result indicated that the number of significantly altered functional connectivities might not be all accounted by the elevated core temperature. However, previous studies reported that core temperature was correlated with cognition and behavior performance deterioration [8], such as reaction time and accuracy [52], [61], working memory capacity and the analysis and retention of visual information [2], [11]. However, the central nervous system and peripheral system alterations were not all the result from elevated temperature. Previous studies demonstrated an inverted U-shaped relationship between performance and temperature, implying that cognition and behavior performances were not linearly correlated with the temperature changes. Some other factors were involved in hyperthermia influences. For example, theories involving the accumulation or depletion of neurotransmitters (such as branched chain amino acids, dopamine, cytokines, etc.) in the central nervous system have been proposed to explain the decline of cognition and behavior performance. Inhibition or promotion of a single neurotransmitter may exert various effects on different brain regions. For example, Bridge et al. [62] found that a high activity in the dopaminergic system was associated with a higher tolerance to exercise during heat exposure. From the perspective of the present results, the alteration of the functional connectivity network might be accounted by the integrated effects of physiological changes instead of purely rectal temperature changes.

Besides consideration of core temperature, we explored the correlations between the altered small world metrics and weight loss, but also failed to find significant correlations. It seemed that the altered functional connectivity pattern during passive hyperthermia might not be accounted for by the weight loss. It seems contradictory that previous studies reported that hyperthermia-induced dehydration was correlated with cognition and behavior performance deterioration [63], [64], such as visual-motor tracking, short-term memory and attention [65]. Possible explanation could be that the weight loss in our study did not reach the extent to which dehydration affected human performance. Previous studies demonstrated significant impairment of cognitive functions at the level of moderate dehydration (mild dehydration: 1–2% lost of body weight, moderate dehydration: 2–5%, severe dehydration: >5%) [66]–[68]. However, the mean weight loss was far from the 2% of weight loss (mean weight loss: 0.51±0.18 kg).

So far, the mechanism of human brain cognition and behavior declining during passive hyperthermia still remains unclear, and several factors were reported to be correlated with this decline, such as the cardiovascular system, neurohormonal activity [4], [5], core body temperature [2], [11] and dehydration [63]. The present study investigated two factors (rectal temperature and weight loss) influences on the human brain, but the potential mechanism still awaits further study.

### Limitations

In the present study, we investigated the passive hyperthermia effects on humans under a level of thermal stress with designated 50°C for approximately 1 h of heat exposure. Previous studies revealed that the extent of both physiological and behavior performance deficits was associated with the extent of heat exposure, including the duration and the intensity of the heat exposure [2]. Therefore, it is necessary to explore the relationship between alteration of functional connectivity in human brains and the extent of heat exposure in a future study. Second, we investigated functional connectivity alteration based on a preselected spherical ROI and AAL atlas. However, previous studies provided evidence of parcellation approach-dependent correlations in the human brain network [69], [70]. Therefore, it is warranted to investigate the hyperthermia-induced disruptions with different approaches in the future. In addition, it is a pity that invasive measurement (such as blood gases, neurotransmitters) is hardly conducted in fMRI studies, therefore our study lacked physiological measurements with their contributions to the altered functional connectivities, such as the cardiovascular system, neurohormonal activity and muscle metabolism [9]. In conclusion, the current investigation showed a disturbed functional connectivity pattern throughout the human brain during and after hyperthermia, providing new evidence for the effects of environmental heat on the CNS.

## Supporting Information

Figure S1 **Schematic of attentional network test.**
(DOC)

Table S1 Anatomical parcellation of the entire brain and their abbreviations used in the paper.
(DOC)

Table S2 Significantly altered functional connectivities between regions divided by the AAL atlas between the HT group and NC group.
(DOC)
