# Supplementary material for: Hyperthermia-Induced Disruption of Functional Connectivity in the Human Brain Network
Source: PLoS One. 2013 Apr 8;8(4):e61157. doi: 10.1371/journal.pone.0061157 (PMC3620175; doi:10.1371/journal.pone.0061157)
Supplement: Figure S1 — Schematic of attentional network test. (DOC) [file pone.0061157.s001.doc]

**Supporting Figure S1.** Schematic of attentional network test


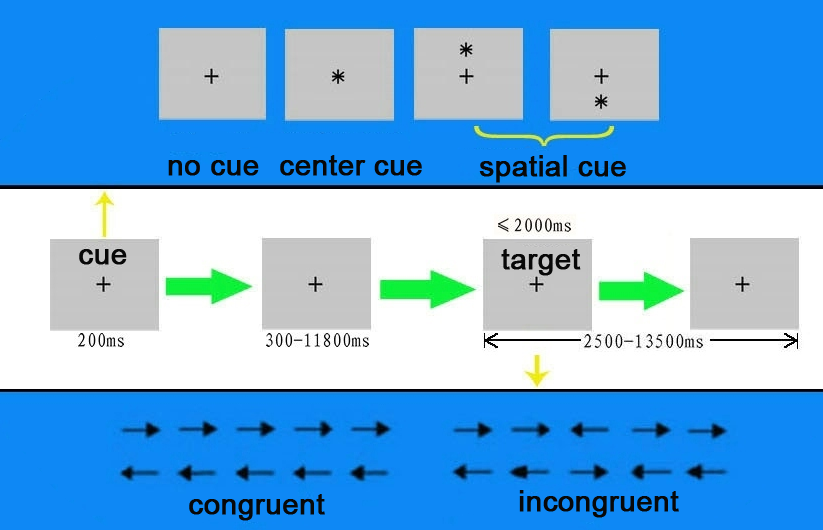


In each trial, a fixation cross appears in the center of the screen all the time. Depending on the cue condition, a cue (none, center, or spatial cue) appears for 200 ms. After a variable duration (300–11800 ms), the target (the center arrow) and flankers of left and right two arrows (congruent or incongruent flankers) are presented until the participant responds with a button press, but for no longer than 2000 ms. After the participant makes a response, the target and flankers disappear immediately and a post-target fixation period lasts for a variable duration (from the onset of the target and the start time of the next trial is between 2500 and 13500 ms).
