# Supplementary material for: Hyperthermia-Induced Disruption of Functional Connectivity in the Human Brain Network
Source: PLoS One. 2013 Apr 8;8(4):e61157. doi: 10.1371/journal.pone.0061157 (PMC3620175; doi:10.1371/journal.pone.0061157)
Supplement: Table S1 — Anatomical parcellation of the entire brain and their abbreviations used in the paper. (DOC) [file pone.0061157.s002.doc]

**Supporting Table S1. Anatomical parcellation of the entire brain and their abbreviations used in the paper**

| **Labels** | **Regions** | **Abbr** | **Labels** | **Regions** | **Abbr** |
| --- | --- | --- | --- | --- | --- |
| 1,2 | Left/Right precentral gyrus | PreCG.L/R | 3,4 | Left/Right superior frontal gyrus, dorsolateral | SFGdor.L/R |
| 5,6 | Left/Right superior frontal gyrus, orbital part | ORBsup.L/R | 7,8 | Left/Right middle frontal gyrus | MFG.L/R |
| 9,10 | Left/Right middle frontal gyrus, orbital part | ORBmid.L/R | 11,12 | Left/Right inferior frontal gyrus, opercular part | IFGoperc.L/R |
| 13,14 | Left/Right inferior frontal gyrus, triangular part | IFGtriang.L/R | 15,16 | Left/Right inferior frontal gyrus, orbital part | ORBinf.L/R |
| 17,18 | Left/Right rolandic operculum | ROL.L/R | 19,20 | Left/Right supplementary motor area | SMA.L/R |
| 21,22 | Left/Right olfactory cortex | OLF.L/R | 23,24 | Left/Right superior frontal gyrus, medial | SFGmed.L/R |
| 25,26 | Left/Right superior frontal gyrus, medial orbital | ORBsup.L/R med | 27,28 | Left/Right gyrus rectus | REC.L/R |
| 29,30 | Left/Right insula | INS.L/R | 31,32 | Left/Right anterior cingulate and paracingulate gyri | ACG.L/R |
| 33,34 | Left/Right median cingulate and paracingulate gyri | DCG.L/R | 35,36 | Left/Right posterior cingulate gyrus | PCG.L/R |
| 37,38 | Left/Right hippocampus | HIP.L/R | 39,40 | Left/Right parahippocampal gyrus | PHG.L/R |
| 41,42 | Left/Right amygdala | AMYG.L/R | 43,44 | Left/Right calcarine fissure and surrounding cortex | CAL.L/R |
| 45,46 | Left/Right cuneus | CUN.L/R | 47,48 | Left/Right lingual gyrus | LING.L/R |
| 49,50 | Left /Right superior occipital gyrus | SOG.L/R | 51,52 | Left/Right middle occipital gyrus | MOG.L/R |
| 53,54 | Left/Right inferior occipital gyrus | IOG.L/R | 55,56 | Left/Right fusiform gyrus | FFG.L/R |
| 57,58 | Left/Right postcentral gyrus | PoCG.L/R | 59,60 | Left/Right superior parietal gyrus | SPG.L/R |
| 61,62 | Left/Right inferior parietal, but supramarginal and angular gyri | IPL.L/R | 63,64 | Left/Right supramarginal gyrus | SMG.L/R |
| 65,66 | Left/Right angular gyrus | ANG.L/R | 67,68 | Left/Right precuneus | PCUN.L/R |
| 69,70 | Left/Right paracentral lobule | PCL.L/R | 71,72 | Left/Right caudate nucleus | CAU.L/R |
| 73,74 | Left/Right lenticular nucleus, putamen | PUT.L/R | 75,76 | Left/Right lenticular nucleus, pallidum | PAL.L/R |
| 77,78 | Left/Right thalamus | THA.L/R | 79,80 | Left/Right heschl gyrus | HES.L/R |
| 81,82 | Left/Right superior temporal gyrus | STG.L/R | 83,84 | Left/Right temporal pole: superior temporal gyrus | TPOsup.L/R |
| 85,86 | Left/Right middle temporal gyrus | MTG.L/R | 87,88 | Left/Right temporal pole: middle temporal gyrus | TPOmid.L/R |
| 89,90 | Left/Right inferior temporal gyrus | ITG.L/R |  |  |  |

The cerebrum is divided into 90 regions (45 in each hemisphere). More details for the parcellation can be seen in [Tzourio-Mazyer et al., 2002].
