# Supplementary material for: Hyperthermia-Induced Disruption of Functional Connectivity in the Human Brain Network
Source: PLoS One. 2013 Apr 8;8(4):e61157. doi: 10.1371/journal.pone.0061157 (PMC3620175; doi:10.1371/journal.pone.0061157)
Supplement: Table S2 — Significantly altered functional connectivities between regions divided by the AAL atlas between the HT group and NC group. (DOC) [file pone.0061157.s003.doc]

**Supporting Table S2.** Significantly altered functional connectivities between regions divided by the AAL atlas between the HT group and NC group

| Labels  Label | Region1  Abbreviation | Classification | Labels  Label | Region2  Abbreviation | Classification | *P* Value |
| --- | --- | --- | --- | --- | --- | --- |
| 3 | SFGdor.L | Prefrontal | 28 | REC.R | Prefrontal | 0.00194 |
| 3 | SFGdor.L | Prefrontal | 87 | TPOmid.L | Temporal | 0.00288 |
| 19 | SMA.L | Other frontal | 28 | REC.R | Prefrontal | 0.00205 |
| 21 | OLF.L | Prefrontal | 83 | TPOsup.L | Temporal | 0.00048 |
| 22 | OLF.R | Prefrontal | 83 | TPOsup.L | Temporal | 0.00134 |
| 23 | SFGmed.L | Prefrontal | 28 | REC.R | Prefrontal | 0.00318 |
| 27 | REC.L | Prefrontal | 35 | PCG.L | Parietal | 0.000014 |
| 27 | REC.L | Prefrontal | 36 | PCG.R | Parietal | 0.00124 |
| 27 | REC.L | Prefrontal | 37 | HIP.L | Temporal | 0.00169 |
| 27 | REC.L | Prefrontal | 43 | CAL.L | Occipital | 0.00442 |
| 27 | REC.L | Prefrontal | 44 | CAL.R | Occipital | 0.00246 |
| 27 | REC.L | Prefrontal | 45 | CUN.L | Occipital | 0.0007 |
| 27 | REC.L | Prefrontal | 46 | CUN.R | Occipital | 0.00301 |
| 27 | REC.L | Prefrontal | 47 | LING.L | Occipital | 0.00378 |
| 27 | REC.L | Prefrontal | 57 | PoCG.L | Parietal | 0.00111 |
| 27 | REC.L | Prefrontal | 65 | ANG.L | Parietal | 0.00017 |
| 27 | REC.L | Prefrontal | 67 | PCUN.L | Parietal | 0.00052 |
| 27 | REC.L | Prefrontal | 69 | PCL.L | Frontal/parietal | 0.005 |
| 27 | REC.L | Prefrontal | 81 | STG.L | Temporal | 0.00205 |
| 27 | REC.L | Prefrontal | 82 | STG.R | Temporal | 0.00197 |
| 27 | REC.L | Prefrontal | 85 | MTG.L | Temporal | 0.00472 |
| 28 | REC.R | Prefrontal | 35 | PCG.L | Parietal | 0.00109 |
| 28 | REC.R | Prefrontal | 44 | CAL.R | Occipital | 0.00479 |
| 28 | REC.R | Prefrontal | 45 | CUN.L | Occipital | 0.0049 |
| 28 | REC.R | Prefrontal | 47 | LING.L | Occipital | 0.00321 |
| 28 | REC.R | Prefrontal | 59 | SPG.L | Parietal | 0.00288 |
| 28 | REC.R | Prefrontal | 65 | ANG.L | Parietal | 0.00212 |
| 28 | REC.R | Prefrontal | 67 | PCUN.L | Parietal | 0.00268 |
| 28 | REC.R | Prefrontal | 85 | MTG.L | Temporal | 0.00421 |
| 35 | PCG.L | Parietal | 39 | PHG.L | Temporal | 0.00352 |
| 39 | PHG.L | Temporal | 65 | ANG.L | Parietal | 0.00157 |
| 39 | PHG.L | Temporal | 66 | ANG.R | Parietal | 0.00473 |
| 39 | PHG.L | Temporal | 82 | STG.R | Temporal | 0.00363 |
| 39 | PHG.L | Temporal | 89 | ITG.L | Temporal | 0.00292 |
| 39 | PHG.L | Temporal | 90 | ITG.R | Temporal | 0.00149 |
| 41 | AMYG.L | Temporal | 66 | ANG.R | Parietal | 0.00495 |
| 43 | CAL.L | Occipital | 87 | TPOmid.L | Temporal | 0.00142 |
| 43 | CAL.L | Occipital | 88 | TPOmid.R | Temporal | 0.0049 |
| 44 | CAL.R | Occipital | 87 | TPOmid.L | Temporal | 0.00193 |
| 44 | CAL.R | Occipital | 88 | TPOmid.R | Temporal | 0.00126 |
| 45 | CUN.L | Occipital | 66 | ANG.R | Parietal | 0.00463 |
| 46 | CUN.R | Occipital | 87 | TPOmid.L | Temporal | 0.00178 |
| 46 | CUN.R | Occipital | 88 | TPOmid.R | Temporal | 0.00295 |
| 47 | LING.L | Occipital | 87 | TPOmid.L | Temporal | 0.00215 |
| 48 | LING.R | Occipital | 87 | TPOmid.L | Temporal | 0.00286 |
| 51 | MOG.L | Occipital | 88 | TPOmid.R | Temporal | 0.00334 |
| 82 | STG.R | Temporal | 90 | ITG.R | Temporal | 0.00232 |
| 83 | TPOsup.L | Temporal | 87 | TPOmid.L | Temporal | 0.00017 |
| 84 | TPOsup.R | Temporal | 87 | TPOmid.L | Temporal | 0.00024 |
| 87 | TPOmid.L | Temporal | 90 | ITG.R | Temporal | 0.00226 |
| **6** | **ORBsup.R** | **Prefrontal** | **36** | **PCG.R** | **Parietal** | **0.00326** |
| **6** | **ORBmid.R** | **Prefrontal** | **36** | **PCG.R** | **Parietal** | **0.00446** |
| **29** | **INS.L** | **Insula** | **33** | **DCG.L** | **Parietal** | **0.00404** |
| **29** | **INS.L** | **Insula** | **42** | **AMYG.R** | **Temporal** | **0.00448** |
| **29** | **INS.L** | **Insula** | **74** | **PUT.R** | **Basal ganglia** | **0.00295** |
| **30** | **INS.R** | **Insula** | **41** | **AMYG.L** | **Temporal** | **0.0046** |
| **30** | **INS.R** | **Insula** | **41** | **AMYG.L** | **Temporal** | **0.00118** |
| **41** | **AMYG.L** | **Temporal** | **75** | **PAL.L** | **Basal ganglia** | **0.00393** |
| **42** | **AMYG.R** | **Temporal** | **75** | **PAL.L** | **Basal ganglia** | **0.00321** |
| **42** | **AMYG.R** | **Temporal** | **76** | **PAL.R** | **Basal ganglia** | **0.0044** |
| **58** | **PoCG.R** | **Parietal** | **63** | **SMG.L** | **Parietal** | **0.00263** |
| **70** | **PCL.R** | **Parietal** | **77** | **THA.L** | **Thalamus** | **0.00494** |
| **70** | **PCL.R** | **Parietal** | **78** | **THA.R** | **Thalamus** | **0.00234** |
| **73** | **PUT.L** | **Basal ganglia** | **74** | **PUT.R** | **Basal ganglia** | **0.00227** |
| **74** | **PUT.R** | **Basal ganglia** | **75** | **PAL.L** | **Basal ganglia** | **0.00393** |

Those significant increased correlations between region1 and region2 were highlighted by bold font while the decreased ones were displayed by normal font.
